# Low-level constitutional mosaicism of *BRCA1* in two women with young onset ovarian cancer

**DOI:** 10.1186/s13053-022-00237-x

**Published:** 2022-09-06

**Authors:** B. Speight, E. Colvin, E. D. Epurescu, J. Drummond, S. Verhoef, M. Pereira, D. G. Evans, M. Tischkowitz

**Affiliations:** 1 East Anglian Medical Genetics Service, Cambridge Biomedical Campus, Box 134, Level 6, Addenbrooke’s Treatment Centre, Addenbrooke’s Hospital, Cambridge, CB2 0QQ UK; 2 Manchester Centre for Genomic Medicine, St Mary’s Hospital, Manchester University NHS Foundation Trust, Manchester Academic Health Science Centre, Oxford Road, Manchester, M13 9WL UK; 3 Oncology & Haematology Directorate, Norfolk & Norwich University Hospital, Colney Lane, Norwich, NR4 7UY UK; 4 Department of Clinical Genetics, Leiden University Medical Center, 2333 ZA Leiden, Netherlands; 5 Division of Evolution and Genomic Sciences, School of Biological Sciences, Faculty of Biology, Medicine and Health, University of Manchester, Manchester Academic Health Science Centre, Manchester, UK; 6 Academic Department of Medical Genetics, University of Cambridge, Cambridge, UK

**Keywords:** *BRCA1*, Mosaicism, De novo, Ovarian cancer, Genetic counselling, Multidisciplinary, Risk management

## Abstract

Germline pathogenic variants in *BRCA1* and *BRCA2* cause hereditary breast and ovarian cancer. The vast majority of these variants are inherited from a parent. De novo constitutional pathogenic variants are rare. Even fewer cases of constitutional mosaicism have been reported and these have mostly been described in women with breast cancer. Here we report low-level constitutional mosaicism identified by Next Generation Sequencing in two women with ovarian cancer. A *BRCA1* c.5074G > A p.(Asp1692Asn) variant detected in the first female at 42 years, classed as likely pathogenic, was found in ~ 52% of reads in DNA extracted from tumour, ~ 10% of reads in DNA extracted from peripheral blood leukocytes and ~ 10% of reads in DNA extracted from buccal mucosa. The second *BRCA1* c.2755_2758dupCCTG p.(Val920AlafsTer6) variant was detected in a female aged 53 years, classed as pathogenic, and was found in ~ 59% of reads in DNA extracted from tumour, ~ 14% of reads in DNA extracted from peripheral blood leukocytes and similarly in ~ 14% of reads in both DNA extracted from buccal mucosa and urine sample. Sanger sequencing confirmed the presence of these variants at a corresponding low level consistent with mosaicism that may not have been detected by this method alone. This report demonstrates the clinical benefit for two women of *BRCA1/BRCA2* germline NGS testing at a depth that can detect low-level mosaicism. As well as informing appropriate treatments, tumour sequencing results may facilitate the detection and interpretation of low-level mosaic variants in the germline. Both results have implications for other cancer risks and for relatives when providing a family cancer risk assessment and reproductive risk. The implications for laboratory practice, clinical genetics management and genetic counselling for constitutional mosaicism of *BRCA1/BRCA2* are discussed.

## Introduction

*BRCA1* and *BRCA2* are the most commonly tested genes in women with epithelial ovarian cancer (EOC) and around 8% of unselected women diagnosed with EOC have a germline pathogenic variant [16]. The vast majority of germline pathogenic variants are inherited from a parent and to our knowledge, only 15 cases of de novo constitutional pathogenic variants have been reported in *BRCA1/BRCA2* [3, 9, 18]. In addition, five cases of constitutional mosaicism have been reported [2, 4, 8–10]. In constitutional mosaicism, an individual has genotypically distinct cells, derived from a single zygote. This occurs because of a post-zygote somatic mutation, early enough in development to affect several tissues.

The first published case report of mosaicism was in a 39 year old woman with bilateral breast cancer and a mosaic *BRCA1* exon 16 deletion in blood and tumour [4]. In 2015, Friedman et al published the first mosaic *BRCA1* sequence variant, detected in a woman with triple negative breast cancer at age 43 years [8]. The variant was present at a frequency of ~ 50% in tumour, but only ~ 5% in DNA from leukocytes, buccal mucosa and non-neoplastic breast tissue. In *BRCA1/BRCA2* testing of over 12,000 unrelated women with breast and/or ovarian cancer in France, Golmard et al. [9] identified four women with de novo variants, including a mosaic *BRCA1* variant in a woman with breast cancer diagnosed at age 41 years [9]. There are two published cases of constitutional *BRCA2* mosaicism described. In 2020, Alhopuro et al reported a woman treated for breast cancer at age 36 years who had a *BRCA2* c.9294C > G, p.(Tyr3098Ter) pathogenic variant in 57% of tumour reads and at a lower, variable level (between 20 and 36% reads) in four different non-neoplastic tissues [2]. Earlier this year, Graf et al described constitutional *BRCA2* mosaicism in a woman treated for ovarian cancer in her mid-50s. Using laser microcapture, Graf et al demonstrated the ability to characterise tumour cell subpopulations and heterogeneity including the presence and ratio of *BRCA1/BRCA2* pathogenic variants [10]. The *BRCA2* c.7795G > T, p.(Glu2599Ter) pathogenic variant was present in their patient at a frequency of 77–78% in ovarian tumour, 17% in non-neoplastic ovarian tissue, and between 21 and 26% in DNA from blood and buccal mucosa. Despite an increase in *BRCA1/BRCA2* germline testing via NGS panels in recent years, mosaicism is still a rarely reported finding.

Constitutional mosaicism arises due to a somatic variant acquired early in embryonic development. If this is a pathogenic variant in *BRCA1* or *BRCA2*, the cells in the body harbouring the variant will have a predisposition to associated cancers. Importantly, mosaicism of a low level, below around 20%, is not consistently detectable by Sanger sequencing, and as there is less likely to be any family history of cancer, mosaicism is likely to be under ascertained.

NGS technology has facilitated the detection of mosaicism more reliably and at much lower levels. NGS testing has moved into a mainstream setting for many cancer types, and clinical genetics services are well placed to support cancer teams in delivering this. Published guidance for health professionals on mosaicism within the field of germline cancer genetics is limited [20]. A mosaic finding on a germline test warrants a referral to a clinical genetics department for genetic counselling and assessment. The rarity of mosaicism and the unknown proportion of body tissues affected make quantifying risks and providing accurate genetic counselling a challenge. Questions raised by a mosaic result include: is the mosaicism confined to the tissue in which cancer has developed, or present within other parts of the body? If present in more than one tissue, does the mosaic level vary and crucially, what is the level in tissue associated with cancer predisposition? Is the same cancer risk management as constitutional carriers relevant for patients with mosaicism? What testing and risk information is appropriate for the patient’s family members? What is the expected reproductive risk, 50% or lower? Many of these questions cannot be answered with certainty but addressing them does form part of the genetic counselling process.

## Case 1 description

A 42-year-old woman of British Indian ethnicity was referred to Cambridge Clinical Genetics service by her local Oncology team. She had been diagnosed with stage 4 high-grade serous ovarian cancer at age 42. At the time of referral, she had undergone six cycles of neoadjuvant chemotherapy (Carboplatin and Paclitaxel), undergone a total hysterectomy and bilateral salpingo-oophorectomy, and had started adjuvant chemotherapy (three cycles of Carboplatin). A family history was obtained and relatively few cancers were reported, except for possible ovarian cancer in one of her paternal aunts (unconfirmed).

**Fig. 1:**
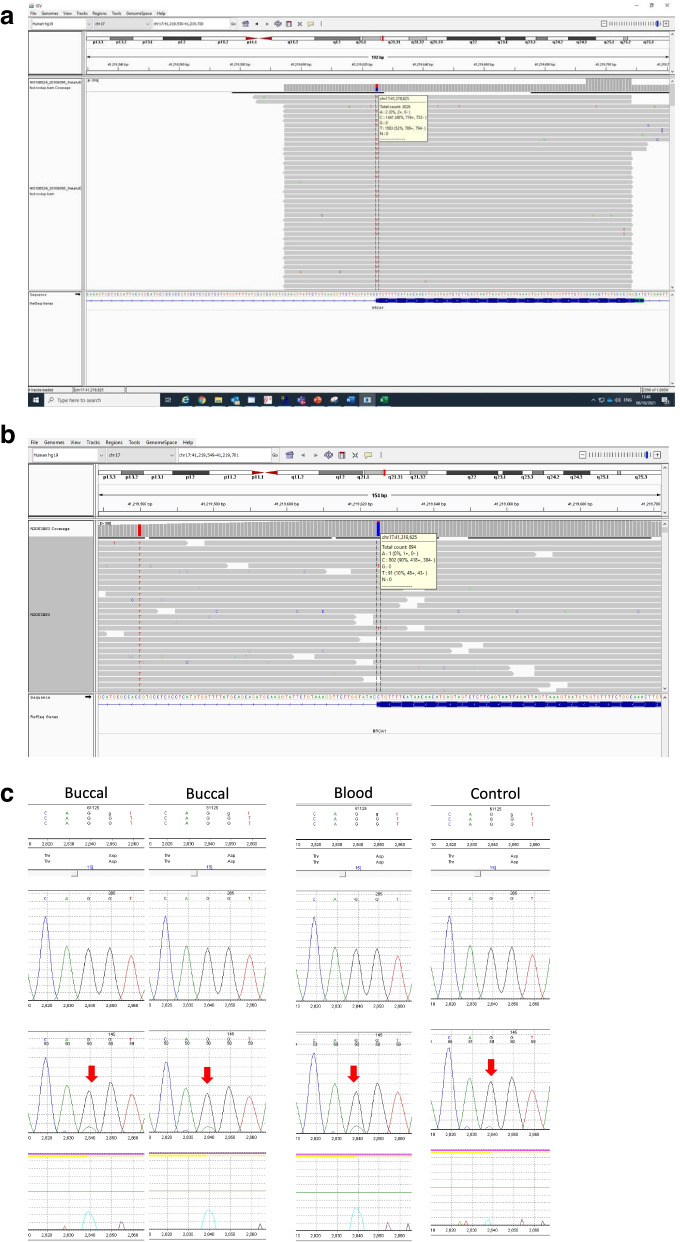
Case 1. Sequencing reads (NGS) from analysis of **a** tumour DNA and **b** blood leukocyte DNA. **c** Sanger sequencing data on DNA extracted from buccal epithelium, a repeat test on buccal epithelium, blood and a control sample

At the time of referral, somatic sequencing on a tumour biopsy and germline sequencing on a blood sample had been done within a mainstream Oncology setting. The blood sample was taken during the time when the patient was receiving neoadjuvant chemotherapy which may have affected the variant allele fraction. NGS analysis with 99.3% coverage with at least 350x coverage depth (mean coverage depth 3353x) detected *BRCA1* c.5074G > A p.(Asp1692Asn) in 52% of reads in the tumour sample (nomenclature according to GenBank accession number NM_007294.3), see Fig. 1a. This variant has previously been reported in the context of hereditary breast and ovarian cancer [17] and has been classified as both likely pathogenic and pathogenic [1, 13]. The variant is absent from population databases [11, 15] and functional studies show that this variant disrupts splicing [7]. NGS analysis of *BRCA1* and *BRCA2* on a blood sample identified the same variant at a lower level, present in approximately 10% of reads, see Fig. 1b. Sanger sequencing confirmed the presence of this variant at a corresponding low level in blood and buccal epithelium consistent with mosaicism, see Fig. 1c. Following referral to the genetics clinic, a test on DNA extracted from a buccal brush sample taken 4 months later during adjuvant chemotherapy also identified the same variant in approximately 10% of reads.

## Case 2 description

A 53-year-old woman diagnosed with ovarian cancer, detected at ultrasound, was referred to the Manchester Clinical Genetics service. She was treated with a total abdominal hysterectomy, bilateral salpingo-oophorectomy, omentectomy and lymph node sampling. She subsequently received chemotherapy (Carboplatin and Paclitaxel). The exact pathological diagnosis was stage 3c high grade mixed serous and endometrioid adenocarcinoma, located in both ovaries, the omentum and the left Fallopian tube, the right tube being unremarkable. Tumour deposits were seen on the serosal surface of the uterus and, the peritoneum, serosa and the right parametrium. The endometrium, myometrium, cervical stroma and epithelium were unremarkable, the lymph nodes showed no evidence of metastasis. The immunological profile was patchy positive for Pax-8 and WT-1, and patchy strong positive for the Estrogen and Progesterone receptors. The family history was negative for other cases of breast cancer or ovarian cancer, but DNA-testing for the *BRCA1* and *BRCA2* genes was offered based on a high grade serous ovarian tumour meeting at least the 10% likelihood threshold defined by UK NICE guidelines [5].

**Fig. 2:**
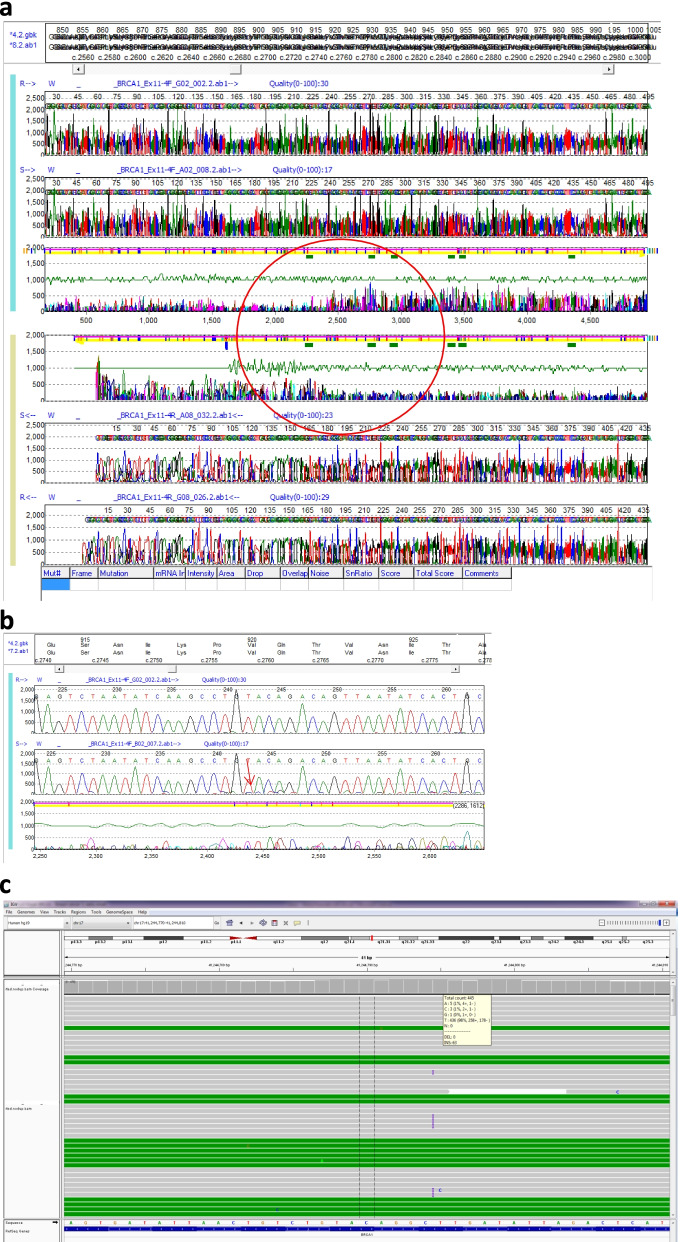
**a** Case 2 - Top and bottom traces = normal control; Two middle traces = patient DNA extracted from blood. Raise in the subtraction indicates the presence of an extra sequencing trace, which in this case corresponds to the mutant allele. **b** Case 2 - Once zoomed in, bottom electropherogram, corresponding to the patient, shows a consistently raised background when compared to the normal control. The lower peaks can be used to read the duplication of the 4 nucleotides CCTG. **c** Case 2 - BAM files for lymphocyte DNA: Shows total read count as 445 and 63 of those indicate an insertion

After referral to the genetics clinic, a blood sample was collected using standard EDTA test tubes. DNA was isolated from the peripheral lymphocytes according to standard procedures (semi-automated procedure on PerkinElmer chemagic MSM1 instrument, using the Chemagic DNA blood kit special (250 preparations from 3 ml blood, article number: CMG-763-C). NGS was carried out on the DNA sample for the full coding sequence and intron-exon boundaries of *BRCA1* and *BRCA2*. A variant was detected at low level, consistent with low level mosaicism. The *BRCA1* c.2755_2758dupCCTG p.(VaI920AIafsTer6), Fig. 2. variant is absent from population databases [11], but is predicted to lead to a premature truncation of the *BRCA1* protein. It is therefore considered as a pathogenic variant. The mosaicism was further explored using a low-level mosaicism NGS pipeline. The estimated level of mosaicism in the lymphocytic DNA was 14% (63 out of 445 reads positive, Fig. 2c, Table 1). NGS sequencing carried out on DNA from a buccal sample and a urine sample yielded mosaic levels in the same order of magnitude (Table 1).

**Table 1:** Case 2 - Summary of samples and variant allele fraction

Source	Depth		%
**Lymphocytes**	≥ 100x	63/445	14
**Urine**	≥ 100x		14
**Saliva**	≥ 100x		14
**Tumour**	8796x		59

The achieved depth on NGS from the representative tumour sample of the ovarian carcinoma tissue was on average 8796X over the complete target coding region, to a minimum depth of 100X (Table 1). The variant was detected in 59% of reads. No other variants were identified. Loss of heterozygosity was observed for 8 informative SNPs in the *BRCA1* gene in the ovarian tumour tissue, in support of *BRCA1* being involved as evidence of a larger ‘second hit’ in tumorigenesis.

## Genetic counselling

Prior to the genetics referral, patient 1 was informed of the *BRCA1* result by her Oncology team. After the referral, a clinic appointment for further genetic counselling was arranged. This was virtual due to the COVID-19 pandemic. Patient-initiated topics covered in the first genetic counselling session included the likelihood of this result as the cause of ovarian cancer and the implications for family members. This led on to establishing a family history record and a discussion of mosaicism as a factor that influences risk to relatives. Prior to information provision, the patient’s understanding of the result and her concept of mosaicism were established. Mosaicism was explained avoiding the use of specialised/medical terminology. Use of visual aids to help understanding was prevented by a transition to telephone-based appointments early in the pandemic. The patient seemed reassured that the chance of wider family members having the variant was unlikely, given the mosaic finding. Revertant mosaicism of a parentally inherited variant has never been reported for *BRCA1*, but could not be excluded since both parents were deceased. Predictive testing in a sibling subsequently gave a negative result. The patient was accepting of the up to 50% chance of having passed it on to her children, due to the possibility the gonadal cell line was derived from embryonic cells containing the variant. Predictive testing in her children was safely deferred based on their young age.

Genetic counsellor-initiated topics covered in the first session with patient 1 and in the results session of patient 2 included *BRCA1*-associated hereditary predisposition to breast and ovarian cancer, risk management options, psychosocial impact of cancer treatment/genetic testing and cancer risk perception. To exclude circulating tumour cells or clonal haematopoiesis as alternative reasons for low-level mosaicism in DNA from peripheral blood leukocytes, further testing of another non-neoplastic tissue was suggested.

Quantifying breast cancer risk associated with a mosaic *BRCA1* variant was complicated by the impossibility of predicting the proportion of breast tissue with the (likely/) pathogenic variant. Theoretically, mosaicism for *BRCA1* may not give rise to risk equivalence of constitutional carriers and there is likely ascertainment bias in the few reported mosaic cases in women with young-onset cancer. Given there is no basis on which to reliably predict a milder phenotype of reduced cancer risks or later-onset diagnosis, evidence-based *BRCA1* risk ranges were provided to both patients [12], alongside standard clinical care for *BRCA1* carriers.

At present, the best individualised breast cancer risk assessments in constitutional *BRCA1/BRCA2* carriers are estimated using a validated programme such as CanRisk [14]. This can take into account carrier status, age, family history, lifestyle, hormonal factors and polygenic risk scores. It was not possible to quantify breast cancer risk for our patient in this way due to insufficient data to inform the model on breast cancer risk after ovarian cancer. Even in a patient who has not had a previous cancer, the CanRisk model, which is otherwise transforming patient care, will not enable individualised risk assessment in the context of a mosaic result. Risk management of further cancers was not described in the three published reports of *BRCA1* mosaicism [4, 8, 9] or the report of *BRCA2* mosaicism in ovarian cancer [10]. In the report of a mosaic *BRCA2* carrier with a history of breast cancer, risk-reducing mastectomy and bilateral salpingo-oophorectomy was offered and taken up following discussion of the genetic result [2].

Based on the *BRCA1*-associated breast cancer risk, annual breast MRI surveillance was offered and organised for both patients. When to discuss risk-reducing mastectomy in *BRCA1/BRCA2* carriers after a diagnosis of advanced stage ovarian cancer can be a challenging area in providing patient-centred genetic counselling [19]. Within the first couple of years after this diagnosis, the focus is usually on active treatment with an intention to prevent or delay relapse. Risk-reducing breast surgery options were not raised in these sessions, with an awareness that the risk/benefit ratio of cancer risk management strategies will change over time.

A plan was made for further testing on a buccal epithelium sample on both patients, and urine sample for patient 2, before arranging follow up appointments. Given the strong concordance in the variant allele frequency of the pathogenic variant between buccal brush sample and blood this was felt to be sufficient evidence to guide clinical management. If the results had been discordant, then fibroblasts from a skin biopsy would have been another source of DNA. At follow up, the result confirming low-level mosaicism in another non-cancer tissue and a review of previous information and topics were covered. Although offspring risks would likely be below 50% as more than one non tumour tissue showed similar rates of mosaicism, tentative likelihoods of 1 in 10 (10% allele frequency) and 1 in 7 for 14% could be used.

## Discussion

These are the first reports of low-level *BRCA1* constitutional mosaicism in patients with ovarian cancer. Both cases provide further evidence for the gynaecological cancer risk from mosaicism of *BRCA1/BRCA2*, which has so far only been reported in a single case of *BRCA2* mosaicism in a woman with ovarian cancer [10]. As genetic testing is increasingly undertaken to guide clinical management, it is likely that more cases of mosaicism will be found, partly as spin off from tumour testing as part of mainstreaming. In parts of the world able to offer women treatment-focussed genetic testing for ovarian cancer, this is often done within a mainstream setting. Streamlining the consent and test process alongside women’s oncological/surgical care can be an efficient way of facilitating high volume genetic testing, and only a minority will require an onward referral to a clinical genetics service. A mosaic result is likely to be unexpected and will be unfamiliar to most healthcare professionals not specialised in genetics. Clinical genetics services have an important role in supporting health professionals within a cancer setting to ensure high quality patient care. Patients are more likely to be confused by a mosaic result, given this possibility is not usually part of information provided at pre-test consenting and it is a more complex result. Ensuring patients have access to a referral for genetic counselling to address questions about the result, other cancer risks and family members is an important part of the patient pathway. The rarity of documented cases of mosaicism makes specific, evidence-based clinical guidance impossible, as does the variable, unpredictable level of mosaicism within an individual. Providing risks to children is usually not straightforward, with the exception of Neurofibromatosis 2, where mosaicism is very common and levels of mosaicism on blood DNA do predict risk in offspring [6]. In the context of genetic testing in a woman with breast or ovarian cancer in which a mosaic *BRCA1/BRCA2* pathogenic variant is found, it currently remains appropriate to discuss risk management options as for constitutional carriers.

## Data Availability

All data generated or analysed during this study are included in this published article.
